# Supplementary material for: The Orexigenic Force of Olfactory Palatable Food Cues in Rats
Source: Nutrients. 2021 Sep 3;13(9):3101. doi: 10.3390/nu13093101 (PMC8471864; doi:10.3390/nu13093101)
Supplement: Supplementary file 1 [file nutrients-13-03101-s001.zip › Figure S1.pdf]

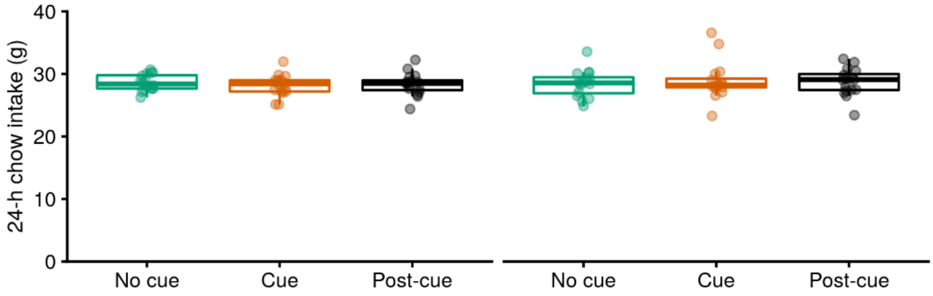

Figure S1. Comparison of the cumulative intake of chow within 24 h following cue removal with that of the baseline and PB cue settings. 24-h cumulative chow intake (g) during the baseline (green), PB cue (orange) and post-cue (black) settings in PB taste-naïve rats (left, n = 16) and PB taste-familiar rats (right, n = 16). Cumulative 24-h chow intake following cue removal was similar to that of the baseline and the cue settings in both PB taste-naïve and familiar rats. For illustration, the thick line always corresponds to the median, boxes show first and third quartiles and whiskers represent minimum and maximum values. Data were analysed by one-way repeated measures ANOVA. No significant differences were observed.
