# Supplementary material for: The Orexigenic Force of Olfactory Palatable Food Cues in Rats
Source: Nutrients. 2021 Sep 3;13(9):3101. doi: 10.3390/nu13093101 (PMC8471864; doi:10.3390/nu13093101)
Supplement: Supplementary file 1 [file nutrients-13-03101-s001.zip › Table S1.pdf]

Table S1. Cumulative chow consumption (1, 3 and 6 h post-cue presentation) in mice presented an olfactory PB cue in the home environment

	**1-h chow intake (g)**	**3-h chow intake (g)**	**6-h chow intake (g)**
**Non-enriched environment** (perforated balls with empty tubes)	0.044 ± 0.025	0.167 ± 0.04	0.363 ± 0.073
**Olfactory PB cue-enriched environment** (perforated balls with PB-filled tubes)	0.137 ± 0.056	0.23 ± 0.051	0.388 ± 0.083

PB, peanut butter; n=10. Data are presented as mean ± SEM and analysed by paired samples *t*-tests. No significant differences were found.
