# Supplementary material for: The Orexigenic Force of Olfactory Palatable Food Cues in Rats
Source: Nutrients. 2021 Sep 3;13(9):3101. doi: 10.3390/nu13093101 (PMC8471864; doi:10.3390/nu13093101)
Supplement: Supplementary file 1 [file nutrients-13-03101-s001.zip › Table S2.pdf]

Table S2. Intake of PB during the 1-h lasting PB tasting conditionings in both rats and mice

	1-h PB intake (g) Day 1	1-h PB intake (g) Day 2	1-h PB intake (g) Day 3
**Rats**	2.01 ± 0.246	3.758 ± 0.268 ***	3.49 ± 0.3
**Mice**	0.297 ± 0.056	0.497 ± 0.074 *	0.56 ± 0.088

PB, peanut butter; rats, n=16; mice, n=10. Symbols indicate significant differences vs. the first PB tasting session at * $p < 0.05$ or *** $p < 0.001$ by paired samples *t*-tests.
