# Supplementary material for: The Orexigenic Force of Olfactory Palatable Food Cues in Rats
Source: Nutrients. 2021 Sep 3;13(9):3101. doi: 10.3390/nu13093101 (PMC8471864; doi:10.3390/nu13093101)
Supplement: Supplementary file 1 [file nutrients-13-03101-s001.zip › Table S3.pdf]

Table S3. Acoustic measurements of the interaction with the perforated ball set up in PB taste-naïve and PB taste-familiar rats

		Minimum (dB)	Average (dB)	Maximum (dB)
**PB taste-naïve rats (n=15)**	No cue	26	32	55
	PB cue	30	42	61
	Δ	**4**	**10**	**6**
**PB taste-familiar rats (n=20)**	No cue	29	37	58
	PB cue	34	50	68
	Δ	**5**	**13**	**10**
**Percentage difference (%Δ)**		**25**	**30**	**67**

PB, peanut butter
